# Genomic insights into plant growth promoting rhizobia capable of enhancing soybean germination under drought stress

**DOI:** 10.1186/s12866-019-1536-1

**Published:** 2019-07-11

**Authors:** Nicholas O. Igiehon, Olubukola O. Babalola, Bukola R. Aremu

**Affiliations:** Food Security and Safety Niche, Faculty of Natural and Agricultural Sciences, Private Mail Bag X2046, North-West University, Mmabatho, 2735 South Africa

**Keywords:** Drought stress, Nitrogen fixation, PGP, Symbiotic establishment, Soybean, Whole genome sequences

## Abstract

**Background:**

The role of soil microorganisms in plant growth, nutrient utilization, drought tolerance as well as biocontrol activity cannot be over-emphasized, especially in this era when food crisis is a global challenge. This research was therefore designed to gain genomic insights into plant growth promoting (PGP) *Rhizobium* species capable of enhancing soybean (*Glycine max* L.) seeds germination under drought condition.

**Results:**

*Rhizobium* sp. strain R1, *Rhizobium tropici* strain R2, *Rhizobium cellulosilyticum* strain R3, *Rhizobium taibaishanense* strain R4 and *Ensifer meliloti* strain R5 were found to possess the entire PGP traits tested. Specifically, these rhizobial strains were able to solubilize phosphate, produce exopolysaccharide (EPS), 1-aminocyclopropane-1-carboxylate (ACC), siderophore and indole-acetic-acid (IAA). These strains also survived and grew at a temperature of 45 °C and in an acidic condition with a pH 4. Consequently, all the *Rhizobium* strains enhanced the germination of soybean seeds (PAN 1532 R) under drought condition imposed by 4% poly-ethylene glycol (PEG); nevertheless, *Rhizobium* sp. strain R1 and *R. cellulosilyticum* strain R3 inoculations were able to improve seeds germination more than R2, R4 and R5 strains. Thus, genomic insights into *Rhizobium* sp. strain R1 and *R. cellulosilyticum* strain R3 revealed the presence of some genes with their respective proteins involved in symbiotic establishment, nitrogen fixation, drought tolerance and plant growth promotion. In particular, *exoX*, *htrA*, *Nif*, *nodA*, *eptA*, *IAA* and siderophore-producing genes were found in the two rhizobial strains.

**Conclusions:**

Therefore, the availability of the whole genome sequences of R1 and R3 strains may further be exploited to comprehend the interaction of drought tolerant rhizobia with soybean and other legumes and the PGP ability of these rhizobial strains can also be harnessed for biotechnological application in the field especially in semiarid and arid regions of the globe.

## Background

The symbiotic interaction between leguminous plants and nitrogen (N) fixing bacteria, generally called rhizobia, has been the focus of research for over 12 decades. Recently, ‘a renewed interest’ in this area of research has been noticed due to its importance in sustainable agriculture, minimizing cost for the agriculturalists, enhancing soil fertility, alleviation of greenhouse-gas emissions [1] and improving plant’s tolerance to drought stress [2].

In addition, the role of soil microorganisms in plant growth, nutrient utilization, drought tolerance as well as biocontrol activity is well known and these beneficial microorganisms inhabit the plant rhizosphere. In the rhizosphere, these microorganisms promote plant growth via ‘direct and indirect mechanisms’ [3]. Additionally, the role of these beneficial microorganisms in biotic and abiotic stresses is gaining relevance and the mechanisms by which they enhance plant tolerance to drought include: Production of ACC deaminase to minimize the quantity of ethylene produced in the roots, microbial exopolysaccharide (EPS), induced systemic resistance and phytohormones production such as indole-3-acetic acid (IAA) [4–7].

Indeed, plant growth can be regulated by ethylene (C_2_H_4_) contents and the biosynthesis of this compound is regulated by biotic and abiotic stressors [8]. In the synthetic pathway of C_2_H_4_ in plants, S-adenosyl methionine (S-AdoMet) is transformed to the immediate precursor of C_2_H_4_ 1-aminocyclopropane-1-carboxylate (ACC) by aminocyclopropane-1-carboxylate synthase (ACS). Under drought stress conditions, plant homeostasis is regulated by C_2_H_4_^,^ leading to decrease in shoot and root growth and even seed germination. Plant ACC is confiscated and disintegrated by ACC deaminase-producing rhizobia to release and supply energy and nitrogen. Thus, the disintegration and consequential removal of ACC by rhizobia alleviate the effects of C_2_H_4_, thereby minimizing plant stress and enhancing plant growth [9]. Therefore single and dual inoculation of plants with ACC-producing rhizobia can result in improved seed germination even under drought stress conditions. In particular, dual inoculation of ACC deaminase producing *Pseudomonas* and *Bacillus* with *Mesorhizobium ciceri* enhanced seed germination, shoot height, root length and seedling fresh weight of chickpea grown under stressed condition when compared to non-inoculated plants [10].

In addition, drought stress affects water availability to plant and water availability regulates the production and utilization of polysaccharides by rhizobia [11]. Example of such polysaccharides is exopolysaccharide (EPS) and production of EPS by rhizobia protects them from harsh conditions, which enhances their survival under such conditions. Amendment of wheat with EPS and catalase producing *Rhizobium leguminosarum* (LR-30), *Rhizobium phaseoli* (MR-2) and *Mesorhizobium ciceri* (CR-30 and CR-39) benefited the plant by improving its growth, drought tolerance index and biomass under drought condition using polyethylene glycol (PEG) 6000 as the drought factor. Thus, there is further need to X-ray the effects of new strains of *Rhizobium* on growth parameters (such as percentage seed germination) of other agricultural crops such as soybean (*G. max* L.) under drought condition stimulated by PEG.

Again, it has been reported that soil bacteria offer benefits to their host plants by suppressing plant pathogens and facilitating nutrient assimilation [4, 12, 13]. In our previous study [2], it was reported that some rhizobacteria mop up the insoluble form of iron from the soil environment and make it available to plants ‘with the aid of siderophore’ [14] and there is an evidence that some plants can use bacterial iron (III)-siderophore complexes for their growth [15] even though the phytorelevance of these complexes is controversial. On the other hand, the removal of iron from the soil by siderophore-producing rhizobia reduces the bioavailability of iron in the root region and consequentially suppresses the growth of fungal pathogens [16, 17].

Similarly, just like siderophore-producing bacteria, some rhizobia contribute to plant growth by helping to mineralize insoluble phosphate compounds to release phosphorus needed for plant growth [18]. Phosphorus in di-calcium phosphate, hydroxyapatite, rock phosphate and tri-calcium phosphate in soil can be released by phosphate solubilizing bacteria such as *Rhizobium*, *Bacillus*, *Burkholderia* and *Agrobacterium* while other rhizospheric rhizobia have the ability to produce indole-acetic acid (IAA) which helps in root elongation and production of lateral roots and root hairs involved in nutrient absorption [19]. Elongation and increase in the number of root produced by plants as a result of IAA production can serve as a survival strategy to plants under drought stress condition and may even contribute in some other ways to plant development. It was reported by [20] that the increased production of IAA by *Bradyrhizobium japonicum* shows that, in addition to plant promotion, the bacterium could have other beneficial traits needed for plants (such as soybean) survival. In short, considering these benefits, the interaction between plants, especially legumes, and rhizobia is key to plant productivity.

Actually, rhizobia-legume symbiotic relationship commences with a molecular dialogue between the partners. The legumes produce flavonoids [21] that elicit the production of Nod factors (lipochitin oligosaccharides), that in turn, stimulate the development of root nodule [22]. Rhizobial species enter and colonize the root nodules where they metamorphose to bacteriods that fix atmospheric N [23]. Admittedly, other bacterial systems are involved in root colonization, efficient nodulation and N-fixation, ‘including surface polysaccharide and secretion systems’ [1, 23, 24]. These processes in addition to PGP and drought tolerance ability of rhizobia are regulated by myriads of genetic components which can further be exploited to gain insights into legume –rhizobial interactions.

Therefore this study was designed to gain genomic insights into selected PGP rhizobia capable of promoting soybean seed germination under drought stress condition.

## Methods

### Source of rhizobial species used in this study

**Fig. 1:**
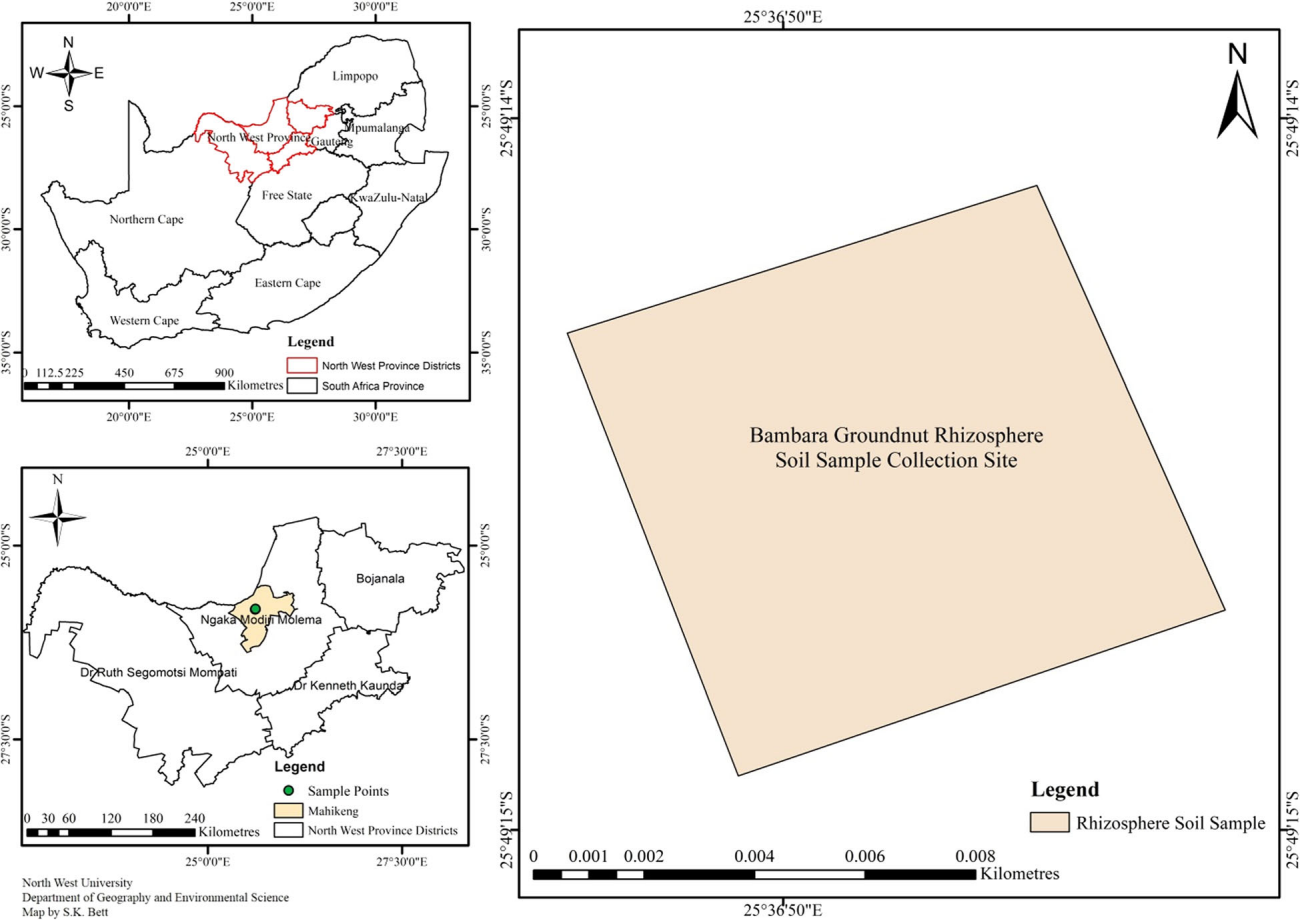
Geographical location of Bambara groundnut rhizospheric soil used for rhizobial species isolation. To the left, the upper sketch represents a map of South Africa showing North-West Province (red sketch) and below is a map of North-West Province accommodating a map of Mahikeng (the light-yellow region) which encompasses Ngaka Modiri Molema district (the green spot) the site of North-West University where Bambara groundnut rhizospheric soil was collected for bacterial isolation. To the right, is a sketch showing Bambara groundnut rhizospheric soil sample collection site

The rhizobial species used in this study were isolated from Bambara groundnut rhizospheric soil at North-West University campus (25.82080S: 025.61382E), Ngaka Modiri Molema District, Mahikeng, North-West Province, South Africa (Fig. 1) and the physicochemical analysis showed that the soil has the following properties: 7.65 pH, 1.62 mg/kg Fe, 24.1 mg/kg Mn, 1.06% organic carbon, 4.01% organic matter, 285 mg/kg K, 397 mg/kg mg and 0.066% total N. The rhizobial species were sequenced by Sanger sequencing technique and identified in our previous study (National Centre for Biotechnology Information – NCBI - database) as *Rhizobium* sp. strain R1 (accession no. MG309875), *Rhizobium tropici* strain R2 (accession no. MG851722), *Rhizobium cellulosilyticum* strain R3 (accession no. MG309874), *Rhizobium taibaishanense* strain R4 (accession no. MG851723) and *Ensifer meliloti* strain R5 (accession no. MG851724).

### ACC deaminase quantification

Rhizobial strains were grown in 5 ml Luria Bertani (LB) at ambient temperature. Then ACC deaminase activity was determined according to method described by [25].

### EPS test

First, rhizobial strains were qualitatively screened for exopolysaccharide production according to the method described by [26] with little modifications. Briefly, sterile Whatman filter paper discs (6 mm in diameter) were aseptically placed in Petri dishes containing nutrient agar and 2 μl of freshly grown cultures of each rhizobial species was directly inoculated on the surfaces of the discs in the plates. The nutrient agar used in this study was amended with 10% sucrose adjusted to pH of 5.5 and 7.5. Upon inoculation, plates were incubated at 28 ± 2 °C, 37 °C and 45 °C for 7 days, 2 days and 1 day at the respective temperatures. Then, EPS production was evaluated on the basis of formation of mucoid colonies around the discs.

Alternatively, the quantity of EPS produced was determined according to the method described by [27] with little modifications. In summary, the four isolates were grown in nutrient broth amended with 5 and 10% PEG 800 to induced drought stress as well as in nutrient broth lacking PEG 800 (0% PEG). Cultures were incubated in a rotary incubator at room temperature for 4 days and were thereafter centrifuged to obtain the supernatant. Three milliliter (3 ml) of cold absolute alcohol was mixed with 5 ml of each rhizobial supernatant and incubated for 12 h at 4 °C. Then EPS was gotten by centrifuging the cold alcohol-rhizobial supernatant mixture at 10000 rpm for 15 min and the resultant supernatants were discarded. Then, the optical density of the EPS that settled at the bottom of the tubes were determined using a spectrophotometer (ThermoSpectronic, Merck) at 490 nm.

### Quantitative determination of siderophore produced by rhizobial species

The quantitative determination of siderophore produced by rhizobial species was determined according to the method described by [28] with little modification. Briefly, freshly grown rhizobial species were inoculated into King B broth (10 g/l glycerine, 20 g/l peptone, 1.5 g/l MgSO_4_) and iron-free succinic acid broth (6 g K_2_HPO_4_, 3 g KH_2_PO_4_, 1 g (NH_4_)_2_SO_4_, 0.2 g MgSO_4_.7H_2_O and 4 g succinic acid) in tubes, while controls were amended with chrome azurol S (CAS) solution and incubated at ambient temperature in a shaker incubator at 120 rpm. Rhizobial broths were centrifuged at 10000 rpm for 10 min. The quantity of siderophore was produced assessed by measuring the optical density of the supernatant at 400 nm.

### Indole-acetic-acid (IAA) test

Quantitative measurement of IAA produced by rhizobia species were determined according to the method described by [18] with little modification. In summary, each rhizobial strain was inoculated in 0.2 L LB broth and incubated in a rotary shaker at ambient temperature for 96 h. One milliliter (1 ml) of the rhizobial broth was centrifuged at 3000 rpm for 30 min, and thereafter, 2 ml of the supernatant was mixed with 2 drops of orthophosphoric acid and 4 ml Salkowski reagent. Optical density of the pink broth was taken at 530 nm using a spectrophotometer (ThermoSpectronic, Merck) and the actual concentration of IAA produced by the rhizobial species was estimated from a standard IAA curve in the range of 0–120 μg/ml.

### Phosphate solubilization test

Phosphate solubilization test was determined as described by [29] with little modification. Pikovskaya’s agar with the following composition per litre was prepared: tricalcium phosphate (5 g), potassium chloride (0.2 g), magnesium sulphate (0.1 g), manganese sulphate (0.0001 g), yeast extract (0.5 g), glucose (10 g), agar (15 g), ammonia sulphate (0.5 g), ferrous sulphate (0.001 g), and the medium was autoclaved at 121 °C for 15 min, after adjusting the pH of the final composition to pH 7.0 using a pH meter. Autoclaved medium was poured on Petri dishes and allowed to solidify. Wells of 8 mm in diameter were made in the medium and inoculated with 25 μl of broth culture of each isolates. Three (3) wells per isolate were used. Plates were incubated at 27 °C for 4 days and a cleared zone around the wells indicated a positive result. Diameters of zones were obtained by measuring the diameter of zone of inhibition minus the diameter of the wells.

### Rhizobial growth response to different temperature

LB broth was prepared according to the manufacturer’s guidelines and autoclaved. Five μl of each rhizobial strain was inoculated in 25 ml of LB broth and gently vortexed. Each rhizobial treatment was replicated 3 times for the different temperature. Inoculated broth was incubated at 28, 35, 45 °C. The O.D (optical density) of the rhizobial growths was taken using a spectrophotometer at 630 nm at days 4, 8, 12, 16 and 20.

### Rhizobial growth response to different pH

LB broth was prepared according to the manufacturer’s guidelines and the pH of the broth was adjusted to acidic (4), neutral (7) and alkaline (10) pH and autoclaved. Five microliters of each rhizobial strain was inoculated in 25 ml of LB broth and gently vortexed. Each rhizobial treatment was replicated 3 times. Inoculated broth was incubated at 28 °C. The OD of the rhizobial growths was taken using a spectrophotometer at 630 nm at days 5, 10, 15 and 20.

### Bacterial growth and preparation

Three (3) of the rhizobial strains were selected for soybean inoculation. Rhizobial spp. were harvested as described by [30] with little modification. Freshly grown cultures of the rhizobial spp. were centrifuged at 5000 rpm for 300 s and the pellets were washed in 0.85% (w/v) normal saline solution and thereafter homogenized in saline solution prior to solution.

### Seed germination test

The colony counts of the rhizobial spp. were 20 × 10^5^ CFU (colony forming unit) ml^− 1^ (for R1 strain), 11 × 10^5^ CFU ml^− 1^ (for R3 strain) and 21 × 10^5^ CFU ml^− 1^ (for R5 strain). Rhizobial suspension (0.5 ml) of each strain was pipetted into Petri dishes containing Whatman filter paper while 0.5 ml of sterile distilled water was transferred to the non-inoculated (control) plates. Soybean seeds (PAN 1532 R) obtained from Agricultural Research Council, South Africa were surface sterilized in 75% alcohol and 1% sodium hypochlorite for 600 s and rinsed in sterile distilled water. Then 30 seeds were place in the Petri dishes containing inoculated filter papers and 4% PEG and the plates were gently swirled. Each treatment was done in triplicate. Parafilm paper was used to seal the plates incubated for 8 days in a growth chamber (GC-300TL, JEIO TECH, Korea) adjusted to 23/16 day/night for periods of 8/16 h night/day at 10,000 light lux for 8 days. The number of germinated seeds was counted afterwards and the percentage seed germination rate was estimated using the following formula:

$$ \mathrm{Percentage}\ \mathrm{seed}\ \mathrm{germination}\ \left(\%\right)=\frac{n}{\mathrm{N}}\mathrm{x}\ 100 $$

Where n is the number of germinated seeds after 8 days and N is the total number of seeds.

### Deoxyribonucleic acid (DNA) extraction for whole genome sequencing

Fresh culture of *Rhizobium* sp. strain R1 and *R. cellulosilyticum* strain R3 was obtained by taking inocula from 50% glycerol and streaking on freshly prepared nutrient agar. Plates were incubated at 28 °C for 4 days and thereafter bacterial DNA was extracted from the fresh isolates using Zymo DNA extraction kit following manufacturer’s the instructions. The purity and concentration was determined by both 1% agarose gel electrophoresis and a NanoDrop spectrophotometer. The DNA extracts were stored at − 20 °C until use. Afterwards, 40 μl of DNA of extract of each bacterium was sent in an ice pack to Molecular Research Laboratory (Mr. DNA), Texas, USA for HiSeq system (illumina) sequencing.

### Sequencing, quality check, trimming and assembly

Following the manufacturer’s instructions, DNA libraries were made from 25 to 50 ng of extracted DNA using KAPA HyperPlus kits (Roche). Upon library preparation, DNA concentration was determined using the Qubit® dsDNA HS Assay Kit (Life Technologies) and average library size was evaluated using Agilent 2100 Bioanalyzer (Agilent Technologies). ‘The workflow combines enzymatic steps and employs minimal bead-based cleanups’. DNA samples were enzymatically degraded into ds DNA fragments and thereafter end repair cum A-tailing were performed to obtain ‘end-repaired, 5’-phosphorylated, 3’-dA-tailed ds DNA fragments.’ Adapter ligation was performed by ligating ds DNA adapters with 3′-dTMP overhangs to 3′-dA-tailed DNA molecules, and thereafter, DNA libraries amplification were performed by using high fidelity and low-bias polymerase chain reaction (PCR). The DNA libraries were then assembled and diluted to 10.5pM and ‘sequenced paired end for 500 cycles using the HiSeq system (Illumina).

Illumina data were extracted and uploaded into Kbase, reads quality was done by performing quality check of the illumina sequence using FastQC (v1.0.4) and low quality sequence and adapter were trimmed off using trimmomatic [31]. Illumina sequence reads were de novo assembled using both SPAdes and ARAST to create contigs.

### Annotation

The genomes of R1 and R3 strains were annotated using Kbase Prokka (V1.12) annotation pipeline and rapid annotation using subsystem technology (RAST) server [32]. The aforementioned systems permit the identification of introns, functional annotations as well as ‘manual curation of gene annotations’. They also possess platforms for metabolic construction with the aid of Kyoto encyclopedia of genes and genomes (KEGG) for comparing sequence using Basic Local Alignment Search Tool (BLAST) and functional comparisons using KEGG and/or FIGfam. The data for R1 and R3 were both given Bioproject number PRJNA496421 while R1 and R3 data were assigned Biosample numbers SAMN10240937 and SAMN10245972 respectively upon submission to the GenBank database. In addition, R1 has SRA Accession number: SRR8060784 and R3 has SRA Accession number: SRR8061690.

### Statistical analyses

Data obtained for the plant growth promoting and seed germination tests were analyzed using Microsoft Excel and Statistical Analysis System (SAS) platforms. Analysis of Variance (ANOVA) was performed for the data followed by Duncan test to determine differences between mean and *P* < 0.05 was considered significant [33, 34]. With respect to sequenced data, mean read length and standard deviation of read length were computed using Kbase pipeline.

## Results

### Plant growth promoting traits of rhizobial species

In this present study, the plant growth promoting traits of rhizobial species were determined.

### Aminocyclopropane-1-carboxylate (ACC) production by rhizobial species

**Fig. 2:**
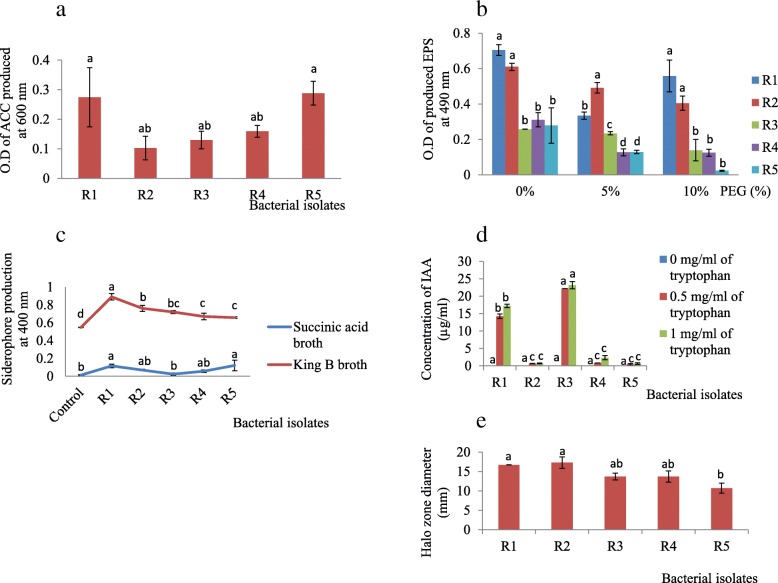
The concentration of **a** ACC (produced under drought stress induced by − 0.30 MPa PEG), **b** EPS, **c** siderophore, **d** IAA and **e** diameter of clear (halo) zones produced by rhizobial species. R1 - *Rhizobium* sp. strain R1, R2 - *Rhizobium tropici* strain R, R3 - *Rhizobium cellulosilyticum* strain R3, R4 - *Rhizobium taibaishanense* strain R4 and R5 - *Ensifer meliloti* strain R5. ACC-1-aminocyclopropane-1-carboxylate, EPS – exopolysaccharide, IAA - Indole-acetic-acid, O.D – optical density. Data represent mean ± SE

With regard to ACC production, R5 strain produced the highest concentration of ACC followed by R1 strain while R2 strain produced the lowest concentration of ACC (Fig. 2a) under stress condition imposed by PEG. These rhizobial strains were further screened for other plant growth promoting traits such as EPS, siderophore production, IAA and phosphate solubilization tests.

### EPS production by rhizobial species

In this study, all rhizobial species produced EPS. In particular, the rhizobial species incubated at 37 °C produced EPS at pH 5.5 and 7.5 but R1 and R4 strains did not produce EPS when incubated at 45 °C while R2 and R3 strains produced EPS under all the environmental conditions considered (Table 1). To be specific, among the rhizobial treatments, R1 strain produced the highest concentrations (0.7 and 0.6 O.D respectively) of EPS at 0 and 10% PEG concentrations and R2 produced the highest EPS at 5% PEG concentration (Fig. 2b) followed by R1 and R3 strains.

**Table 1:** Qualitative response of bacteria towards exopolysaccharide (EPS) assay

Bacterial strain	Response at pH 5.5			Response at pH 7.5		
	28 °C ± 2	37 °C	45 °C	28 °C ± 2	37 °C	45 °C
R1	+	+	–	+	++	–
R2	+	++	+	+	++	++
R3	+	++	+	+	++	++
R4	+	++	–	+	++	++
R5	ND	++	+	ND	+	++

Legend: + = positive, - = negative, + + = strongly positive, ND =Not determined, R1= *Rhizobium* sp. strain R1, R2 = *Rhizobium tropici* strain R2, R3 = *Rhizobium cellulosilyticum* strain R3, R4 = *Rhizobium taibaishanense* strain R4 and R5 = *Ensifer meliloti* strain R5

### Siderophore production by rhizobial species

The ability of rhizobial species to produce siderophore in different media (succinic acid broth and King B broth) showed that all the rhizobial species produced more siderophore in King B broth compared to succinic acid broth (Fig. 2c). R1 strain produced the highest concentration of 0.9 O.D in King B broth while R1 and R2 strains produced more siderophore than R4 and R5 strains in succinic acid broth (Fig. 2c). Conversely, the control treatments amended with CAS solution showed the lowest values for both media.

### IAA production by rhizobial species

The ability of rhizobial species to produce IAA under different tryptophan concentrations revealed higher concentrations of IAA production by R1 and R3 strains. In particular, R3 strain produced the highest concentrations of IAA (22.19 and 23.155 μl) at 0.5 and 1 mg/ml of tryptophan respectively, followed by R1 strain, but the lowest concentrations were produced by R5 strain (Fig. 2d).

### Phosphate solubilization by rhizobial species

As regards phosphate solubilization, R2 strain comparatively showed a bigger halo-zone in Pikovskaya’s agar with a mean diameter of 17.3 mm while R1 strain showed a mean diameter of 16.7 mm. The diameter of the halo-zone produced by R5 strain was lowest in this study (with a mean value of 10.7 mm). Nevertheless, R3 and R4 strains produced halo zones with the same diameter (13.7 mm) (Fig. 2e).

### Rhizobial growth response under environments with different temperatures

**Fig. 3:**
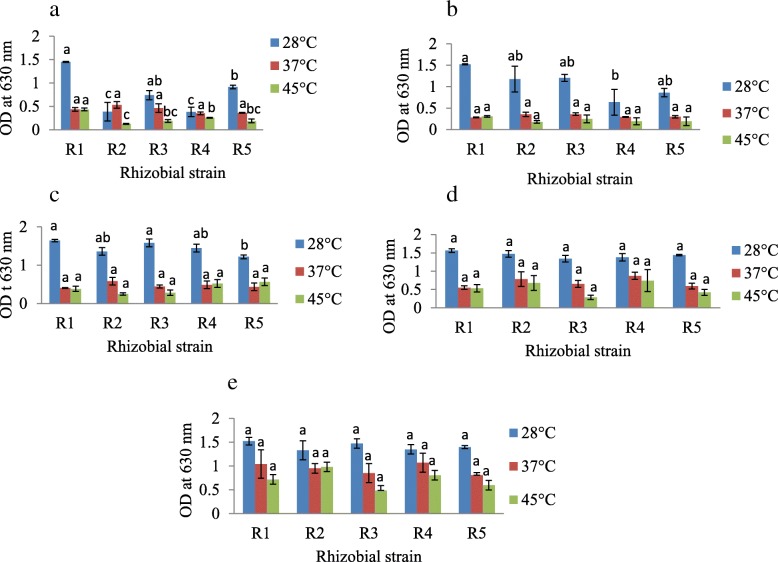
Bacterial growth response to different environmental temperatures on day **a** 4, **b** 8, **c** 12, **s** 16 and **e** 20. R1 - *Rhizobium* sp. strain R1, R2 - *Rhizobium tropici* strain R2, R3 - *Rhizobium cellulosilyticum* strain R3, R4 - *Rhizobium taibaishanense* strain R4 and R5 - *Ensifer meliloti* strain R5. Data represent mean ± SE

Considering the response of rhizobial species towards different environmental temperatures, we observed that R1 strain showed the highest growth at 28 °C as depicted by O.D values throughout the experimental period. However, at 45 °C, rhizobial growth response modulated throughout the experimental period. As an illustration, R1 strain showed the highest O.D values of 0.4 and 0.3 on day 4 and 8 respectively but R5 and R4 strains showed the highest growths of 0.564 and 0.7 O.D corresponding to day 12 and 16 while R2 strain had the highest O.D value of 0.98 on day 20. The same pattern of rhizobial growths was observed at 37 °C (Fig. 3a, b, c, d and e).

**Fig. 4:**
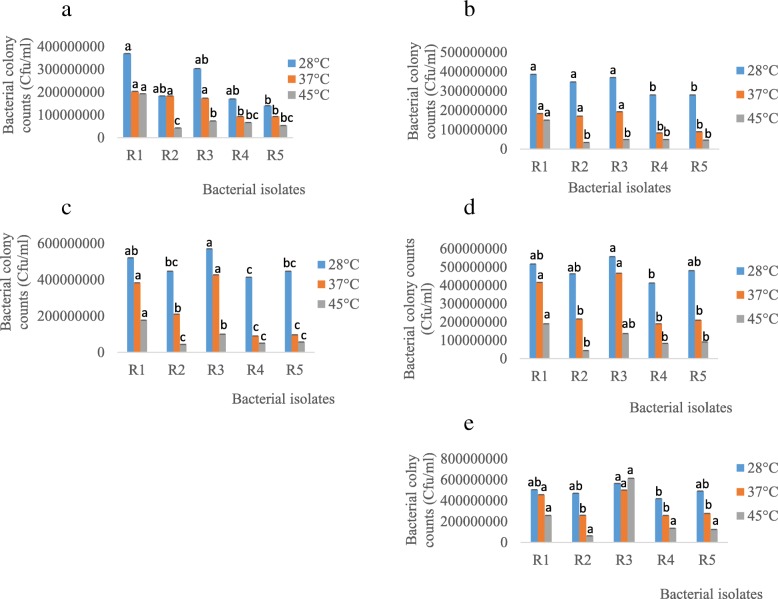
Bacterial growth response to different environmental temperatures on day **a** 4, **b** 8, **c** 12, **d** 16 and **e** 20. R1 - *Rhizobium* sp. strain R1, R2 - *Rhizobium tropici* strain R2, R3 - *Rhizobium cellulosilyticum* strain R3, R4 - *Rhizobium taibaishanense* strain R4 and R5 - *Ensifer meliloti* strain R5. Data represent mean ± SE

From the plate count method, R1 (193333333.3 Cfu/ml) had the highest count followed by R3 (73333333.3 Cfu/ml) at 45 °C on day 4 (Fig. 4a). Similarly, R1 had the highest counts on day 8, 12 and 16 while R3 showed the highest growth on day 20 at 45 °C (Fig. 4b, c, d, and e). This further indicates that R1 and R3 are relatively more tolerant to heat.

### Rhizobial growth response under environments with different pH

**Fig. 5:**
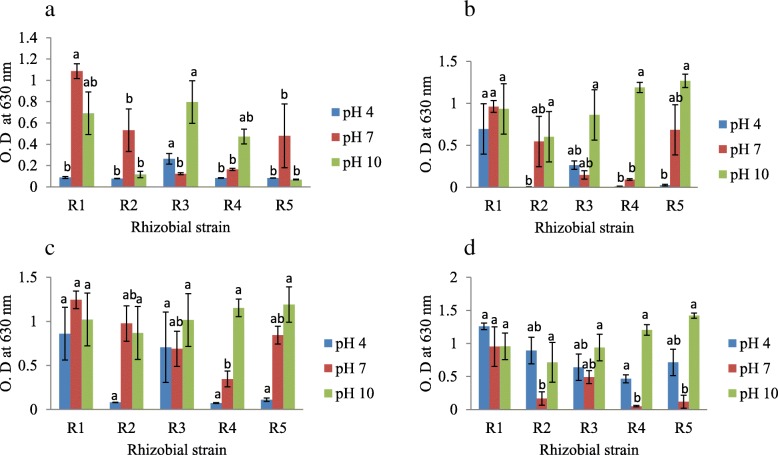
Rhizobial growth response to different environmental pH on day **a** 5, **b** 10, **c** 15 and **d** 20. R1 - *Rhizobium* sp. strain R1, R2 - *Rhizobium tropici* strain R2, R3 - *Rhizobium cellulosilyticum* strain R3, R4 - *Rhizobium taibaishanense* strain R4 and R5 - *Ensifer meliloti* strain R5. Data represent mean ± SE

From the spectrophotometric method, R1 strain tended to showed better growth at a pH of 4 at the onset of rhizobial growth response to pH experiment (Fig. 5a) and later decreased as the experiment progressed. However, R1 strain responded more positively at pH 7 throughout the experimental sampling period (Fig. 5a, b, c and d), but R3 grew better at pH 10 on day 5 (Fig. 5a) while R5 strain was more abundant (with a cell biomass of 0.79 O.D) on day 20 under this pH condition (Fig. 5d).

**Fig. 6:**
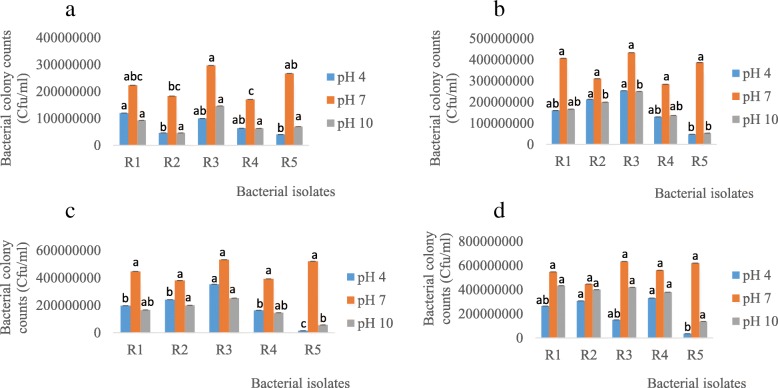
Rhizobial growth response to different environmental pH on day **a** 5, **b** 10, **c** 15 and **d** 20. R1 - *Rhizobium* sp. strain R1, R2 - *Rhizobium tropici* strain R2, R3 - *Rhizobium cellulosilyticum* strain R3, R4 - *Rhizobium taibaishanense* strain R4 and R5 - *Ensifer meliloti* strain R5. Data represent mean ± SE

On the other hand, from the plate count method, R1 (120, 000000 Cfu/ml) had the highest count followed by R3 (100000000 Cfu/ml) at pH 4 on day 5 (Fig. 6a). On the contrary, at the same pH, R3 (146666666.7 Cfu/ml) had the highest count followed by R1 (93333333.33 Cfu/ml) (Fig. 6a). Similarly, R3 had the highest counts on day 10 and 15 at both extreme pH (4 and 10) while R4 and R1 showed the highest growth on day 20 at pH 4 and 10 respectively (Fig. 6b, c, d).

### Soybean seed germination

**Fig. 7:**
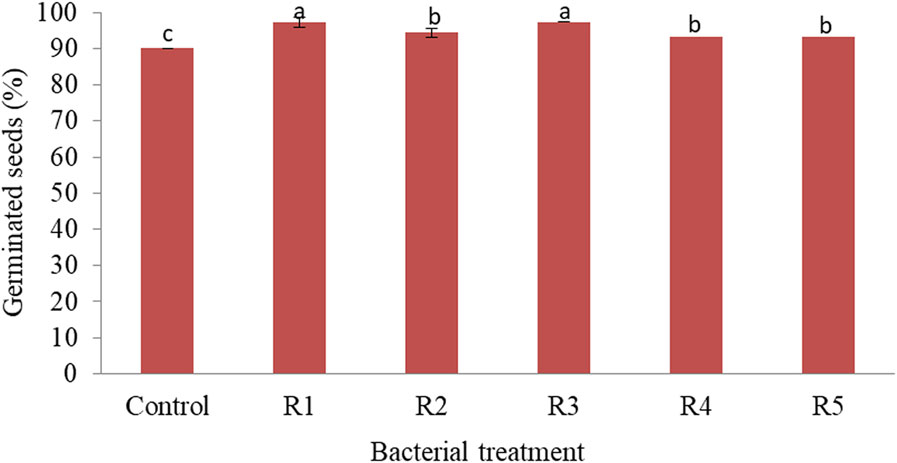
Percentage of soybean seeds inoculated with rhizobial species that germinated in Petri dishes. R1 - *Rhizobium* sp. strain R1, R3 - *Rhizobium cellulosilyticum* strain R3 and R5 - *Ensifer meliloti* strain R5. Data represent mean ± SE

The effects of R1, R2, R3, R4 and R5 inoculation on soybean seeds germination under drought stressed condition imposed by 4% PEG revealed that R1 and R3 strains had a better effect on soybean germination with a percentage seed germination of 97.3% each when compared to R2, R3 and R5 strains with a percentage seed germination of 94.4, 93.3 and 93.3% respectively. However, the non-inoculated (control) experiment showed the lowest percentage seed germination of 90% (Fig. 7). Thus, whole genome sequencing was performed for R1 and R3 strains in order to gain genomic insights into some of the functional genes that may be involved in drought tolerance, symbiotic establishment as well as plant survival and growth promotion.

### Genomic overview of R1 and R3 strains

Prior to illumina sequencing, DNA concentration of 35.6 ng/μL, DNA library concentration of 81.60 ng/μL with an average size of 647 bp were generated for R1 strain while R3 strain yielded 50.2 ng/μL, 90.40 and 661 bp corresponding to DNA concentration, final DNA library concentration and average library size (Table 2).

**Table 2:** DNA final library concentration and average library size

Rhizobial species	DNA concentration (ng/μL)	Final DNA library concentration (ng/μL)	Average library size (bp)
R1	35.6	81.60	647
R3	50.2	90.40	661

Legend: R1 - *Rhizobium* sp. strain R1 and R3 - *Rhizobium cellulosilyticum* strain R3

Upon de novo assembly, R1 strain was found to have 17,408,810 reads with a mean length of 201.15 and a total of 5773 contigs. The number of genes predicted was 29842 with a GC content of 61.91%. The N_50_ value of 936 was obtained for the scaffold. On the other hand, R3 strain had 17,794,094 reads with a mean read length of 214.18 and 129 contigs. The genome size of the strain was 4,114,542 with guanine-cytosine (GC) content off 43.59%. The N_50_ value of 57294 was obtained for the scaffold.

### EPS producing genes

**Fig. 8:**
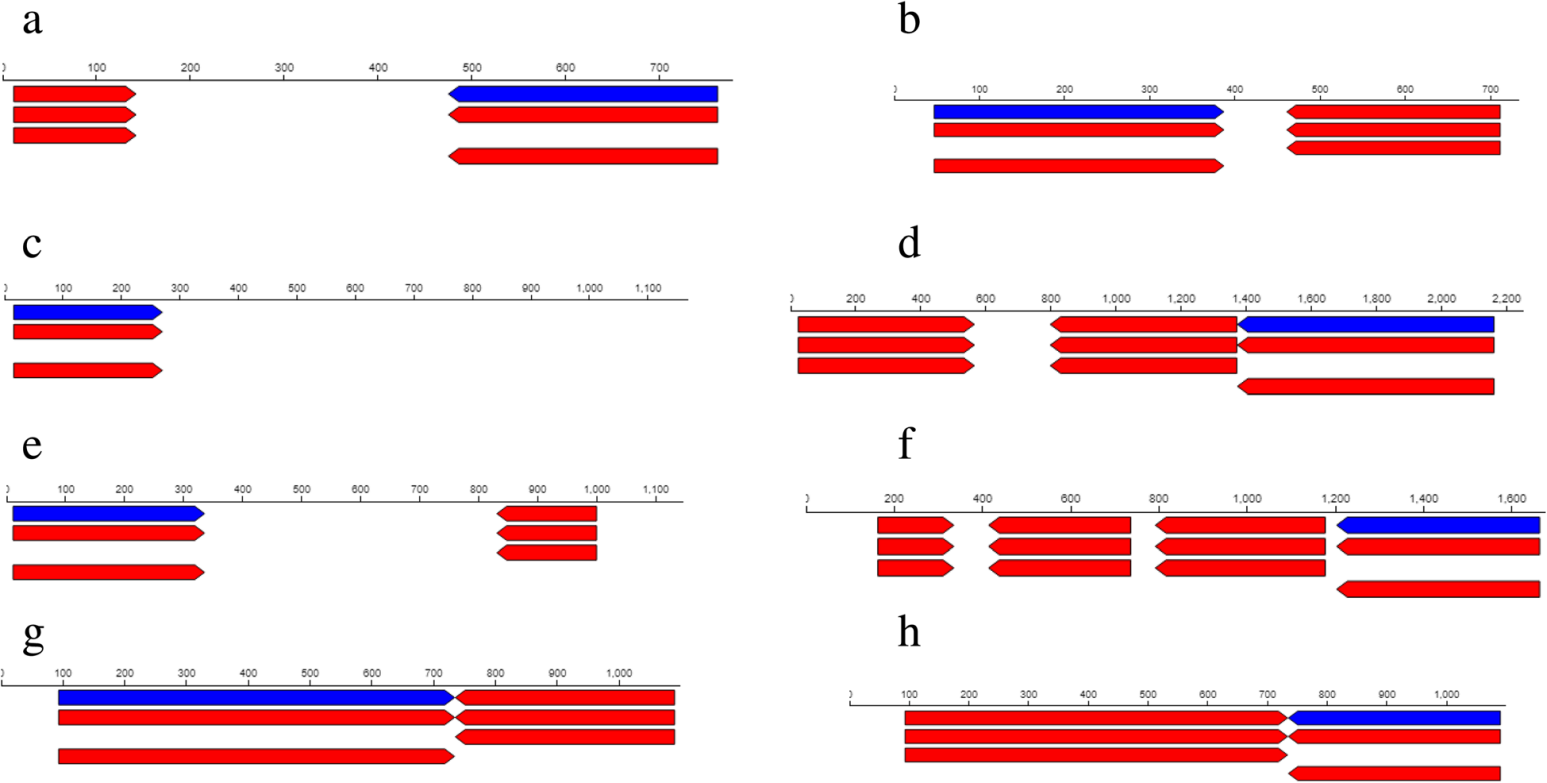
Feature context of **a** Signal transduction histidine-protein kinase BaeS **b** Exodeoxyribonuclease III **c** Extracellular serine protease **d** Microbial serine proteinase **e** Cysteine desulfurase SufS **f** Cysteine desulfurase IscS **g** putative MFS-type transporter YcaD and **h** Riboflavin transporter depicting gene names in the genome map of R1 strain. The blue bars represent the gene locations

Whole genome sequencing revealed 78 *exoX* genes in R1 strain and 99 *exoX* genes in R3 strain and these genes are responsible for the production of exopolysaccharide in the bacterial species. Two (2) of the 78 *exoX* genes found in R1 strain code for signal transduction histidine-protein kinase BaeS and exodeoxyribonuclease III proteins with the corresponding baeS_1, 2.7.13.3 and xthA 3.1.11.2 aliases (Table. 3). The location of the signal transduction histidine-protein kinase gene was between 474 and 762 contigs (Fig. 8a) and that of BaeS and exodeoxyribonuclease III proteins gene was between 47 and 389 contigs (Fig. 8b). On the other hand, of the 99 *exoX* genes found in R3 strain, 2 encode signal transduction histidine-protein kinase ArlS and response regulator aspartate phosphatase J with arlS 2.7.13.3 and rapJ_2 3.1 aliases respectively (Table 4). Also, the location of the signal transduction histidine-protein kinase ArlS gene was between 3,808–5,173 contigs (Fig. 10a) while that of response regulator aspartate phosphatase J gene was between 26,133–27,255 contigs (Fig. 10b).

**Table 3:** Selected stress tolerance, symbiotic and plant growth promoting functional genes found in the genome of *Rhizobium* sp. strain R1

Feature ID	Type	Function	Aliases	Start	Length	Location
Exo X						
JKFNCFJO_00944	gene	Signal transduction histidine-protein kinase BaeS	baeS_1, 2.7.13.3	762	288	Contig: NODE_1409_length_750_cov_2.449333 474–762 (− Strand)
JKFNCFJO_01811	gene	Exodeoxyribonuclease III	xthA, 3.1.11.2	47	342	Contig:NODE_2692_length_718_cov_3.898329 47–389 (+ Strand)
HtrA						
JKFNCFJO_06099	gene	Extracellular serine protease	3.4.21.-	16	255	Contig: NODE_11432_length_1141_cov_2.421560 16–271 (+Strand)
JKFNCFJO_02038	gene	Microbial serine proteinase	aspA, 3.4.21.-	2164	792	Contig:NODE_3069_length_2224_cov_3.164568 1,372–2,164 (−strand)
Nif genes						
JKFNCFJO_00231	gene	Cysteine desulfurase SufS	sufS, 2.8.1.7	11	327	Contig: NODE_344_length_1119_cov_2.287757 11–338 (+Strand)
JKFNCFJO_04114	gene	Cysteine desulfurase IscS	iscS, 2.8.1.7	1,664	462	Contig: NODE_6900_length_1667_cov_2.808638 1,202–1,664 (−Strand)
Nod A						
JKFNCFJO_00001	gene	putative MFS-type transporter YcaD	ycaD_1	93	642	Contig: NODE_1_length_1071_cov_2.464052 93–735 (+Strand)
JKFNCFJO_00002	gene	Riboflavin transporter	ribN	1,091	357	Contig: NODE_1_length_1071_cov_2.464052 734–1,091 (− Strand)
Siderophore						
JKFNCFJO_00628	gene	Catecholate siderophore Receptor Fiu	fiu	1,424	762	Contig: NODE_980_length_1476_cov_2.882791 662–1,424 (− Strand)
JKFNCFJO_07774	gene	2,3-dihydro-2,3-dihydroxybenzoate dehydrogenase	dhbA, 1.3.1.28	59	513	Contig: NODE_18194_length_629_cov_2.839428 59–572 (+ Strand)
IAA						
JKFNCFJO_03956_CDS	CDS	Isoaspartyl peptidase	iaaA, 3.4.19.5	704	168	Contig: NODE_6586_length_704_cov_2.566761 536–704 (− Strand
JKFNCFJO_03956	gene	Isoaspartyl peptidase	iaaA, 3.4.19.5	704	168	Contig: NODE_6586_length_704_cov_2.566761 536–704 (−Strand)
EptA gene						
JKFNCFJO_06547	gene	UDP-N-acetylmuramate--L-alanyl-gamma-D-glutamyl-meso-2,6-diaminoheptandioate ligase	mpl, 6.3.2.45	782	1,365	Contig: NODE_12707_length_2871_cov_2.986416 782–2,147 (+ Strand)
JKFNCFJO_05747	gene	Phosphoethanolamine transferase EptA	eptA_3, 2.7.-.-	42	534	Contig: NODE_10489_length_1026_cov_2.685185 42–576 (+Strand)

**Table 4:** Selected stress tolerance, symbiotic and plant growth promoting functional genes found in the genome of *R. cellulosilyticum* strain R3

Feature ID	Type	Function	Aliases	Start	length	Location
Exo X gene						
LKJIOFBO_00637	gene	Signal transduction histidine-protein kinase ArlS	arlS, 2.7.13.3	5,173	1,365	NODE_12_length_14500_cov_30.995518 3,808–5,173 (−Strand)
LKJIOFBO_01604	gene	Response regulator aspartate phosphatase J	rapJ_2, 3.1.-.-	26,133	1,122	NODE_32_length_40656_cov_31.105864 26,133–27,255 (+Strand)
htrA						
LKJIOFBO_03117	gene	Serine protease Do-like HtrA	htrA_1, 3.4.21.107	6,151	1,203	Contig:NODE_64_length_16935_cov_30.627930 6,151–7,354 (+ Strand
LKJIOFBO_04119	gene	Serine protease Do-like HtrA	htrA_2, 3.4.21.107	24,021	1,362	Contig:NODE_203_length_47982_cov_29.809929 24,021–25,383 (+ Strand)
Nif gene						
LKJIOFBO_00593	Gene	Cysteine desulfurase IscS	iscS_2, 2.8.1.7	40,060	1,146	NODE_11_length_78586_cov_30.601507
LKJIOFBO_01424_CDS	CDS	Putative cysteine desulfurase NifS	nifS, 2.8.1.7	72	264	NODE_29_length_80092_cov_31.155697
Nod A						
LKJIOFBO_00005	gene	Beta-N acetylglucosaminidase	lytD, 3.2.1.96	4,582	2,643	Contig: NODE_1_length_64013_cov_32.129894 4,582–7225 (+Strand)
LKJIOFBO_00006	gene	Teichoic acid poly (ribitol-phosphate) polymerase	tarL, 2.7.8.-			Contig: NODE_1_length_64013_cov_32.129894 7,268–9,128 (− Strand)
Siderophore						
LKJIOFBO_02039	gene	putative siderophore transport system permease protein YfiZ	yfiZ_1	14,769	1,002	Contig: NODE_38_length_39997_cov_29.245344 14,769–15,771 (+ Strand)
LKJIOFBO_02038	gene	putative siderophore-binding lipoprotein YfiY	yfiY	14,769	978	Contig: NODE_38_length_39997_cov_29.245344 13,660–14,638 (− Strand)
IAA						
LKJIOFBO_01515	gene	Inner membrane protein YiaA	yiaA	11,965	264	Contig: NODE_30_length_44008_cov_32.195396 11,701–11,965 (− Strand)
LKJIOFBO_02665	gene	tRNA dimethylallyltransferase	miaA, 2.5.1.75	10,955	945	Contig: NODE_48_length_27057_cov_29.404369
EptA						
LKJIOFBO_03929	gene	Heptaprenyl diphosphate synthase component 1	hepS, 2.5.1.30	8,080	765	Contig: NODE_102_length_18176_cov_29.706535 7,315–8,080 (− Strand)
LKJIOFBO_00595	gene	Septation ring formation regulator EzrA	ezrA	43,376	1,689	Contig: NODE_11_length_78586_cov_30.601507 41,687–43,376 (− Strand)

### High-temperature stress response genes

Again, 5 *htrA* and 6 *htrA* genes were found in R1 and R3 strains. *HtrA* genes are involved in tolerance to high temperature and therefore the survival and growth of R1 and R3 strains observed at 45 °C (Figs. 3a, b, c, d, e & 4a, b, c, d, e) may be due to the high temperature tolerant proteins produced by these microorganisms (Tables 3 and 4).

Notably, 2 of the *htrA* genes found in R1 strain are responsible for the production of extracellular serine protease and microbial serine proteinase (Table 3) and they were located between 16 and 271 and 1,372–2,164 contigs respectively (Fig. 8c, d). In the same way, R3 strain had 2 genes coding for serine protease Do-like HtrA (Table 4) but with different contigs locations (Fig. 10c, d).

### Nitrogen fixing genes

Nitrogen fixing (*nif*) genes are involved in the conversion of atmospheric N to the form that can be utilized by plants. Two (2) of the *nif* genes noticed in R1 strain are involved in the production of cysteine desulfurase SufS and cysteine desulfurase IscS with the corresponding sufS 2.8.1.7 and iscS 2.8.1.7 aliases (Table 3). The locations of these genes were between 11 and 338 contigs for cysteine desulfurase SufS gene and 1,202–1,664 (Fig. 8e) for cysteine desulfurase IscS gene (Fig. 8f). Regarding R3 strain, 2 of its *nif* genes are involved in the production of cysteine desulfurase IscS and Putative cysteine desulfurase NifS proteins (Table 4). These protein producing genes with different aliases also had different contigs locations (Fig. 10e, f).

### Nodulation genes

Nodulation genes play a key role in nodule formation in plant roots where *Rhizobium* species establish symbiosis with host plants. As for R1 strain, 23297 *nodA* genes were found and 2 of the genes possess putative MFS-type transporter YcaD and riboflavin transporter protein potential (Table 3) with contigs locations between 93 and 735 and 734–1,091 respectively (Fig. 8g, h). On the contrary, 2 of the 12242 genes found in R3 strain code for Beta-N acetylglucosaminidase and Teichoic acid poly (ribitol-phosphate) polymerase situated between 4,582–7225 and 734–1,091 contigs respectively (Fig. 10g, h).

### Siderophore-producing genes

**Fig. 9:**
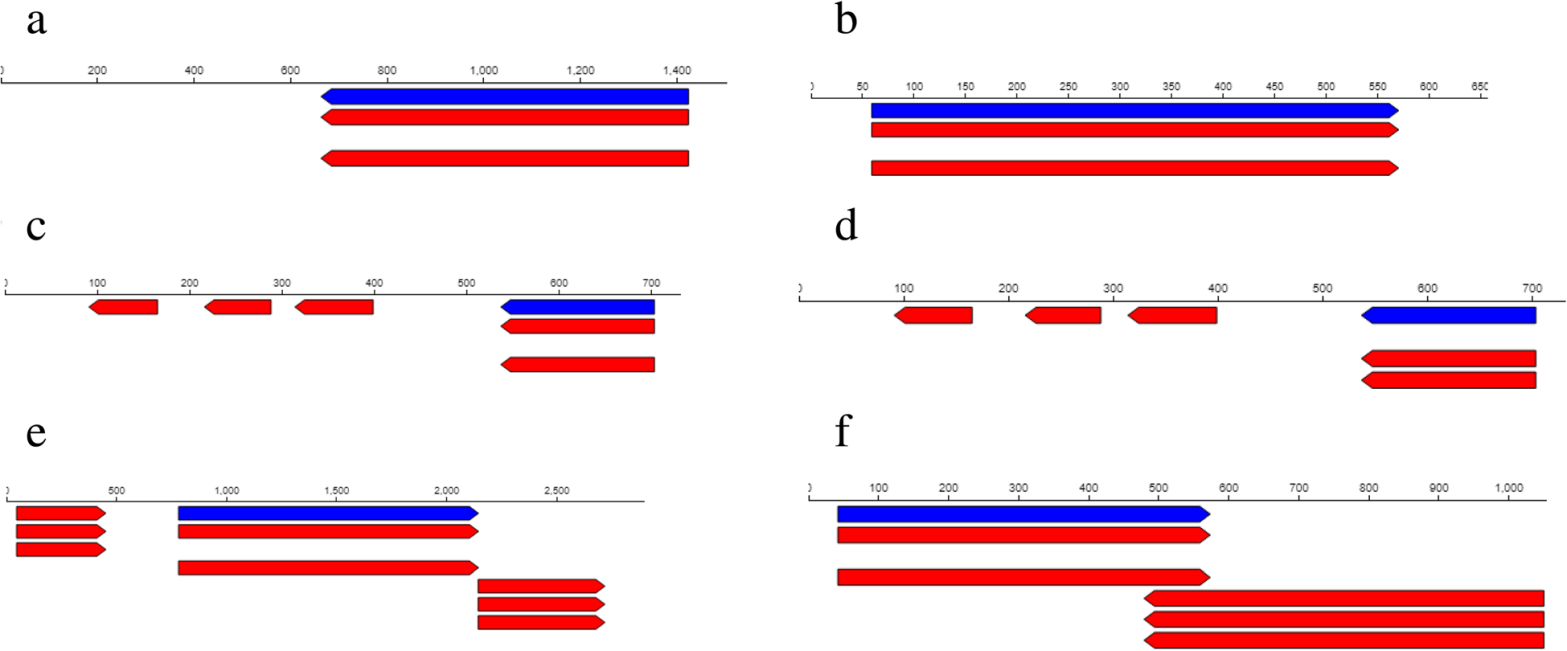
Feature context of **a** Catecholate siderophore Receptor Fiu **b** 2,3-dihydro-2,3-dihydroxybenzoate dehydrogenase **c** Isoaspartyl peptidase **d** Isoaspartyl peptidase **e** UDP-N-acetylmuramate--L-alanyl-gamma-D-glutamyl-meso-2,6-diaminoheptandioate ligase **f** Phosphoethanolamine transferase EptA depicting gene names in the genome map of R1 strain. The blue bars represent the gene locations

At the same time, R1 strain was found to have 13 siderophore-producing genes and 2 of the genes evidently produce Catecholate siderophore Receptor Fiu and 2, 3-dihydro-2,3 dihydroxybenzoate dehydrogenase. The Catecholate siderophore Receptor Fiu gene had fiu aliases while 2, 3-dihydro-2, 3 dihydroxybenzoate dehydrogenase gene had dhbA 1.3.1.28 aliases (Table 3) situated between contigs 662–1,424 (Fig. 9a) and 59–572 (Fig. 9b) within the genome. With respect to R3 strain, it had 33 siderophore-producing genes and 2 of the genes and their respective contigs locations within the genome are shown in Table 4 and Fig. 11a, b accordingly.

### IAA producing genes

Unlike R3 strain, 1 CDS and 1 IAA producing-gene were found in R1 strain with the biological function of Isoaspartyl peptidase (Table 3) and they were both located within 536–704 contigs (Fig. 9c, d). But, R3 strain had 6 IAA-producing genes and 2 of the genes produce inner membrane protein YiaA and tRNA dimethylallyltransferase proteins with the corresponding yiaA and miaA 2.5.1.75 aliases. The locations of their respective gene were also different within the genome (Fig. 11c, d). In reality, proteins produced by these genes can be involved in root elongation and lateral root production in plants.

### Low-pH stress response genes

As a matter of fact, R1 and R3 strains were also found to possess genes that are involved in tolerance to low pH environments and one of them reported in this study is collectively called *eptA.* Indeed, R1 strain had 12 *eptA* genes and 2 of the genes were found to have the biological functions shown in Table 3. R3 strain also had several of the *eptA* genes and 2 of the genes had heptaprenyl diphosphate synthase component 1 and septation ring formation regulator EzrA biological functions (Table 4). These genes were found to be located within different contigs locations in the genome of R1 (Fig. 9e, f) and R3 (Fig. 11e, f) strains.

## Discussion

In this present study, the plant growth promoting traits of rhizobial species were determined. To be specific, the ACC experiment revealed the production of ACC by the rhizobial species (Fig. 2a) but the highest concentration of ACC was produced by R5 strain followed by R1 strain while the lowest concentration of ACC was produced by R2 strain. The production of ACC by these microorganisms shows that they have the potential to increase plant tolerance to drought stress since it was reported by [25] that application of ‘ACC deaminase-producing microorganisms’ into water stressed soil environments can reduce stress in the local plants by minimizing stress triggered by C_2_H_4_. Indeed, a study carried out by [35] showed that ACC deaminase-producing *Pseudomonas* species partially eradicated the detrimental effects of water stress on pea (*Pisum sativum* L.) growth and/or productivity.

In a study performed by [27], bacterial tolerance to water stress conditions was characterized by EPS production and therefore in this study, rhizobial tolerance to drought stress was determined by their ability to produce EPS in medium amended with different concentrations of PEG (drought stress stimulant). Based on the qualitative results, we found that environmental stresses such as pH and temperature stimulated the production of EPS. In particular, R2, R3 and R4 strains produced EPS under the different pH and temperature conditions (Table 1). Quantitatively, our findings showed that R1 strain was more effective in EPS production under severe drought condition (10% PEG) while R2 strain produced more EPS at 5% PEG (Fig. 2b). Other rhizobial species produced EPS at different concentrations in this study. Indeed, EPS production by bacteria protect them from water stress, heavy metals and other environmental stresses [27, 36], and therefore, it is possible for these rhizobial species to survive, multiply and harness other plant growth promoting traits when applied under drought conditions - as evident in the soybean germination experiment (Fig. 7) even in a complex soil environment in the field.

Indeed, siderophore production by different microorganisms has been reported by many researchers [37, 38]. In this present study, the ‘maximum siderophore production’ was found in King B broth. These quantitative results for siderophore production further validate the qualitative plate test for siderophore production for these rhizobial species in our previous study (data not shown). However, the results of this study contradict the findings of [28] who reported maximum siderophore production by *Pseudomonas* species in succinic acid broth.

The rhizobial species used in this study showed other plant growth promoting traits. In particular, all the rhizobial species produced IAA but at different concentrations. This is in agreement with the report that IAA production can differ among different bacterial species, which can be influenced by culture condition, nutrient availability and growth stage [19]. In addition, bacteria from plant rhizosphere are more effective producers of IAA than those from bulk soil [39]. In this study, we observed that R3 strain was more efficient in producing IAA at both concentrations of tryptophan (Fig. 2d) and this could be the reason for the high root biomass observed in soybean treated with this species in our previous study (data not shown), since IAA production has been implicated in root elongation and development of lateral roots [19].

Another strategic mechanism that can be used by rhizospheric bacteria to support the growth of agricultural crops lies in their capacity to solubilize phosphate, and it has been stated that phosphates always occur in bound forms in the soil [27]. Indeed, we found that R2 strain was more effective in solubilizing tri-calcium phosphate in Pikovskaya’s agar followed by R1 strain, but R3 and R4 strains showed similar phosphate solubilizing potential while R5 strain demonstrated the least ability to degrade phosphate as shown in Fig. 2e. Thus, these rhizobial species have the tendency to solubilize bound phosphates and make them available for plant uptake in the soil.

Additionally, rhizobial growth response towards different environmental temperatures showed that these species possess the capacity to thrive and survive at relatively high temperature (45 °C). It has been shown that the optimal growth temperature for many rhizobial species is 25–30 °C [40], which agrees with our findings, since in this study, rhizobial species grew best at 28 °C. The ability of these species to grow and survive at 37 and 45 °C indicate that they may be able to help agricultural crops such as soybean to survive in most tropical countries currently facing drought and/or high temperature problems and further contradict the report of [41, 42] that soybean rhizobial species grow poorly at 40 °C and none of the species is ‘able to grow’ at 42 °C. On the contrary, some rhizobial species capable of nodulating common bean (*Phaseolus vulgaris*) can survive at 47 °C, although they do not have the ability to form nodules at such high temperatures [43]. Other rhizobial species from *Phaseolus vulgaris* are able to survive and remain infective at 40 °C [41].

Additionally, the ability of rhizobial species to grow and survive at different pH was experimented in this study, since real-life biotechnological application of microbial inoculants under drought condition in the field would demand that these species should possess the capacity to adapt to pH fluctuations inherent in complex soil ecosystem. Thus, all the rhizobial species in this current study were able to grow and survive in acidic (pH 4), neutral (pH 7) and alkaline environments (pH 10) (Fig. 5a, b, c, d & 6 a, b, c, d), indicating that these species possibly have broad environmental adaptability with respect to environmental pH. Such trait can help these microorganisms to function actively (without interference) in their symbiotic interactions with crops, since it was reported by [44] that nodulation of faba bean treated with *Rhizobium leguminosarum* was inhibited significantly by soil alkalinity.

The use of R1, R2, R3, R4 and R5 strains for in vitro enhancement of soybean germination under drought condition stimulated by 4% PEG showed that R1 and R3 strains were able to effectively enhance the germination of soybean than R2, R4 and R5 strains. This result showed that R1 and R3 strains were able to enhance the germination of soybean more effectively than the other 3 strains. This finding is in agreement with the results of [45] who reported that ‘ACC deaminase – producing fluorescent pseudomonads’ improved canola (*Brassica napus* L) seed germination under osmotic stress.

Regarding genomic insights into R1 and R3 strains, annotation of R3 genome revealed the presence of 99 different genes (*exo* genes) responsible for EPS production. One of the genes with aliases *yjcG* had the biological function of putative phosphoesterase while *ArlS* gene had the biological function of signal transduction histidine-protein kinase. These EPS genes are known to empower microorganisms to survive under harsh environmental conditions [11]. Although, it has been reported that EPS production is a survival strategy needed by microorganisms under drought stress condition, [1] reported that production of EPS is connected to acid tolerance. In reality, EPS has also been produced by *Agrobacterium tumefaciens* and *S. meliloti* in acidic environments [46–48].

**Fig. 10:**
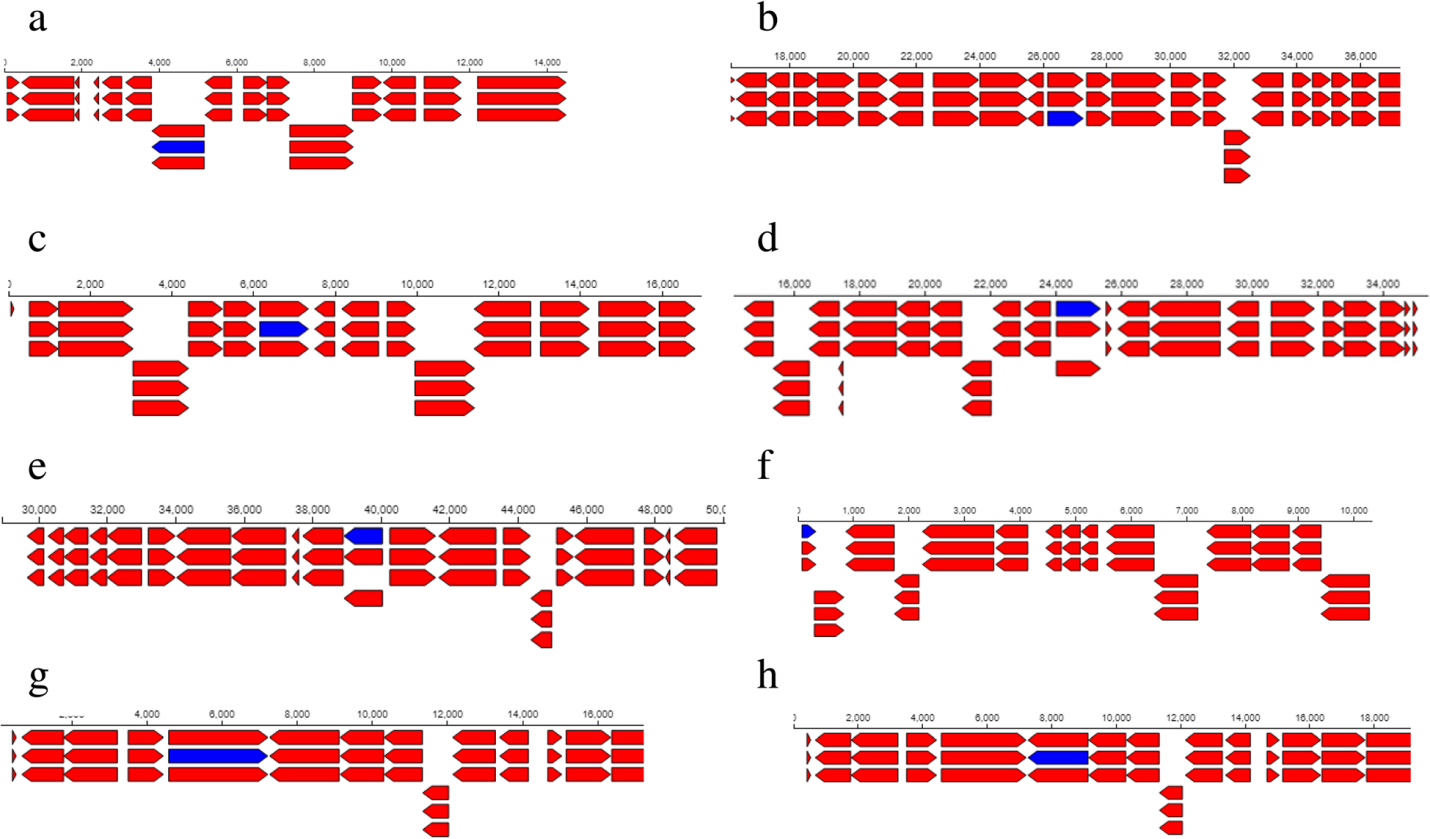
Feature context of **a** Signal transduction histidine-protein kinase ArlS **b** Response regulator aspartate phosphatase J **c** Serine protease Do-like HtrA **d** Serine protease Do-like HtrA **e** Cysteine desulfurase IscS **f** Putative cysteine desulfurase NifS **g** Beta-N acetylglucosaminidase **h** Teichoic acid poly (ribitol-phosphate) polymerase depicting gene names in the genome map of R3 strain. The blue bars represent the gene locations

Under certain circumstances, surface polysaccharides such as EPS, capsular polysaccharides (CPS), β-1, 2-glucans and lipopolysaccharides (LPS), which are essential molecules for symbioses establishment [49, 50] might have other functions such as defense against antimicrobial substances and oxidative stress [51–53]. Different types of *exo* genes have been reported [54], but in this study, we are reporting only *exoX* genes produced by R1 and R3 strains. It was also reported by [54] that *exoX* gene regulates the production of exopolysaccharide (such as succinoglycan) by a ‘new *Rhizobium meliloti*’. The 2 *exoX* genes reported for R1 strain were located between contigs 474–762 and 47–389 (Fig. 8a, b) with signal transduction histidine-protein kinase BaeS and exodeoxyribonuclease III biological functions respectively (Table 3). Similarly, R3 strain had its 2 *exo*X genes located between 3,808–5,173 and 26,133–27,255 (Fig. 10a, b) with signal transduction histidine-protein kinase ArlS and response regulator aspartate phosphatase J functions respectively (Table 4).

As previously mentioned, R1 and R3 strains were able to survive and grow at 45 °C (Fig. 3a, b, c, d, e & 4 a, b, c, d, e) and this trait is thought to be essential for their success as semiarid and/or arid inoculant species [1, 55, 56]. A number of proteins are induced upon exposure to relatively high temperatures and these proteins are generally termed heat shock proteins (HSPs). A couple of HSPs were present in R1 strain and *htrA* genes were found in both R1 and R3 strains. Specifically, 5 *htrA* and 14 *htrA* genes were correspondingly found in R1 and R3 strains. Besides, many different *htrA* homologues are similarly found in the genome of other bacteria [57]. However, it was reported that alterations in one ‘*htrA* paralogue’ of *Brucella abortus* and *S. meliloti* had only a little impact on growth at high temperatures [58, 59], perhaps as a result of functional redundancy. Furthermore, [1] also reported *htrA* gene as one of the components of heat shock response in *Rhizobium tropici* CIAT 899 and *Rhizobium* sp. PRF 81. In reality, besides high temperatures, some HSPs also offer protection against other stressful conditions such as the DnaK machinery which protects against salt stress [60] and even the HtrA that proffers fortification against oxidative damage [59]. Among the *htrA* found in the rhizobial strains in this present study are the R1 strain extracellular serine protease and microbial serine proteinase *htrA* (Table 3) and the R3 strain serine protease Do-like *htrA* and serine protease Do-like *htrA* (Table 4). Again, both *htrA* genes for R1 strain (Fig. 8c, d) and R3 strain (Fig. 10c, d) have their unique contigs locations within the genome.

R1 and R3 strains harbored nitrogen fixing (*nif*) genes with 6 different biological functions and 2 of the genes are presented in Tables 3 and 4 respectively. With respect to location, iscS_1, 2.8.1.7 gene was located between contigs 38, 300–39,443 (Fig. 10e) while *nifS* was located between contigs 72–336 (Fig. 10f) in R3 strain but the *nif* genes in R1 were found at contigs locations (Fig. 8e, f) different from that of R3 strain. IscS has the biological function of providing sulphur for the synthesis of iron-sulphur cluster in vitro; nevertheless, in vivo role of IscS in iron-sulphur formation is yet to be established [61]. Studies of the *Azotobacter vinelandii* nitrogen fixation gene cluster revealed that there are activities that enhance the effectiveness of iron-sulphur cluster assembly [62]. To be specific, study of *nifS* led to the detection that the protein produced by IscS gene is a pyridoxal 59-phosphate-haboring cysteine desulfurase that helps to transfer the sulfur moiety from cysteine to cysteinyl active site of *nifS* leading to the formation of enzyme-bound persulfide [63]. After reduction and incorporation of an iron source, the sulphur can be released and effectively integrated into the iron-sulphur protein cluster of the nitrogenase enzyme complex [63].

However, R1 strain was found to possess other types of *nif* genes such as *nifW*, *nifN*, *nifQ* etc. with negative (−) strands and these genes were found to have different biological products (Table 5). Also, R3 strain was also found to possess different nitrogen fixing genes with almost similar products, for instance, *nifX* and *nifQ* both with positive (+) strands with biological products similar to nitrogenase FeMo-cofactor synthesis molybdenum delivery protein NifQ (Table 6).

**Table 5:** *Nif* - genes of *Rhizobium* sp. strain R1

Genome ID	Accession	Annotation	Feature Type	Start	End	Length	Strand	PATRIC genus-specific families (PLfams)	PATRIC cross-genus families (PGfams)	AA Length	Gene Symbol	Product
379,344	379.344.con.0068	PATRIC	CDS	55853	56356	504	–		PGF_00025976	167	nifQ	Nitrogenase FeMo-cofactor synthesis molybdenum delivery protein NifQ
379,344	379.344.con.0068	PATRIC	CDS	56356	57762	1407	–		PGF_06674514	468	nifB	Nitrogenase FeMo-cofactor synthesis FeS core scaffold and assembly protein NifB
379,344	379.344.con.0068	PATRIC	CDS	57927	59501	1575	–		PGF_03973235	524	nifA	Nitrogenase (molybdenum-iron)-specific transcriptional regulator NifA
379,344	379.344.con.0068	PATRIC	CDS	63101	63361	261	–		PGF_01958698	86	nifW	Nitrogenase-stabilizing/protective protein nifW
379,344	379.344.con.0068	PATRIC	CDS	66771	67241	471	–		PGF_00025961	156	nifX	Nitrogenase FeMo-cofactor carrier protein NifX
379,344	379.344.con.0068	PATRIC	CDS	67228	68613	1386	–		PGF_00945843	461	nifN	Nitrogenase FeMo-cofactor scaffold and assembly protein NifN
379,344	379.344.con.0068	PATRIC	CDS	68624	69997	1374	–	PLF_379_00003254	PGF_00025964	457	nifE	Nitrogenase FeMo-cofactor scaffold and assembly protein NifE
379,344	379.344.con.0068	PATRIC	CDS	70319	70981	663	–		PGF_00025962	220	nifX	Nitrogenase FeMo-cofactor carrier protein NifX
379,344	379.344.con.0068	PATRIC	CDS	71250	72812	1563	–		PGF_00025953	520	nifK_1	Nitrogenase (molybdenum-iron) beta chain (EC 1.18.6.1)
379,344	379.344.con.0068	PATRIC	CDS	72868	74319	1452	–	PLF_379_00003331	PGF_00025951	483	nifK_1	Nitrogenase (molybdenum-iron) alpha chain (EC 1.18.6.1)
379,344	379.344.con.0068	PATRIC	CDS	74333	75214	882	–		PGF_00025954	293	nifH	Nitrogenase (molybdenum-iron) reductase and maturation protein NifH

**Table 6:** *Nif* – and *Nif* ^*+*^ genes of *R. cellulosilyticum* strain R3

Genome ID	Accession	Annotation	Feature Type	Start	End	Length	Strand	FIGfam ID	PATRIC genus-specific families (PLfams	PATRIC cross-genus families (PGfams)	AA Length	Gene Symbol	Product
379,345	379.345.con.0001	PATRIC	CDS	5136	5657	522	–	FIG00017434			173	nifE	[NiFe] hydrogenase HoxFUYH(E) maturation factor HoxW
379,345	379.345.con.0001	PATRIC	CDS	3068	3526	459	+	FIG00004977		PGF_00025668	152	nifX	NifX-associated protein
379,345	379.345.con.0001	PATRIC	CDS	649	1251	603	–		PLF_44937_00001956	PGF_03973235	200	nifA	Nitrogenase (molybdenum-iron)-specific transcriptional regulator NifA
379,345	379.345.con.0002	PATRIC	CDS	37348	38454	1107	–				368	nifA	Nitrogenase (molybdenum-iron)-specific transcriptional regulator NifA
379,345	379.345.con.0003	PATRIC	CDS	4051	4725	675	+	FIG00021623		PGF_00025976	224	nifQ	Nitrogenase FeMo-cofactor synthesis molybdenum delivery protein NifQ
379,345	379.345.con.0003	PATRIC	CDS	42478	43125	648	+		PLF_222_00002287	PGF_00052664	215	nifQ	Similar to nitrogenase FeMo-cofactor synthesis molybdenum delivery protein NifQ
379,345	379.345.con.0003	PATRIC	CDS	84935	85576	642	–	FIG00021623	PLF_222_00002287	PGF_00052664	213	nifQ	Similar to nitrogenase FeMo-cofactor synthesis molybdenum delivery protein NifQ
379,345	379.345.con.0003	PATRIC	CDS	40839	41456	618	+	FIG00021623	PLF_222_00002287	PGF_00052664	205	nifQ	Similar to nitrogenase FeMo-cofactor synthesis molybdenum delivery protein NifQ
379,345	379.345.con.0003	PATRIC	CDS	102443	103093	651	+	FIG00021623	PLF_222_00002287	PGF_00052664	216	nifQ	Similar to nitrogenase FeMo-cofactor synthesis molybdenum delivery protein NifQ

Also, we found 23297 and 12242 *nodA* genes in R1 and R3 strains respectively but only 2 of the genes are reported for each strain in this study. Particularly, we observed *NodA* genes encoding putative MFS-type transporter YcaD and riboflavin transporter in R1 strain (Table 3) and Beta-N acetylglucosaminidase and Teichoic acid poly (ribitol-phosphate) polymerase in R3 strain (Table 4). The location of putative MFS-type transporter YcaD was between contigs 93–735 (Fig. 8g), riboflavin transporter was between contigs 734–1091 (Fig. 8h), Beta-N acetylglucosaminidase was between contigs 4582–7225 (Fig. 10g) and Teichoic acid poly (ribitol-phosphate) polymerase was between contigs 7268–9128 (Fig. 10h). It is a common knowledge that *Nod* genes help in the formation of nodules, the site of nitrogen fixation by nitrogen fixing bacteria such as *Rhizobium* species. Moreover, it was suggested that the kind of ‘Nod factor acyl group attached by *NodA* can contribute to the determination of host range’ [1, 64]. *NodA* gene was also reported by [1] as one of the nodulation genes found in *Rhizobium* species.

In addition, R1 strain was noticed to have gene with + strand responsible for the production of nodulation protein N (Table 7) while R3 possesses genes, one with + strand and another with – strand, that can produce protein translocase subunit SecD/protein translocase subunit SecF and swarming motility protein SwrC (Table 8).

**Table 7:** *Nod +* gene of *Rhizobium* sp. strain R1

Genome ID	Accession	Annotation	Feature Type	Start	End	Length	Strand	PATRIC genus-specific families (PLfams)	PATRIC cross-genus families (PGfams)	AA Length	Gene Symbol	Product
379,344	379.344.con.0013	PATRIC	CDS	117351	117827	477	+		PGF_12857966	158		Nodulation protein *N*

**Table 8:** *Nod* ^*−*^ *and Nod +* gene of R. cellulosilyticum strain R3

Genome ID	Accession	Annotation	Feature Type	Start	End	Length	Strand	FIGfam ID	PATRIC genus-specific families (PLfams)	PATRIC cross-genus families (PGfams)	AA Length	Gene Symbol	Product
379,345	379.345.con.0001	PATRIC	CDS	344702	346915	2214	+			PGF_00038653	737		Protein translocase subunit SecD / Protein translocase subunit SecF
379,345	379.345.con.0004	PATRIC	CDS	209310	212468	3159	–			PGF_00055361	1052		Swarming motility protein SwrC

**Fig. 11:**
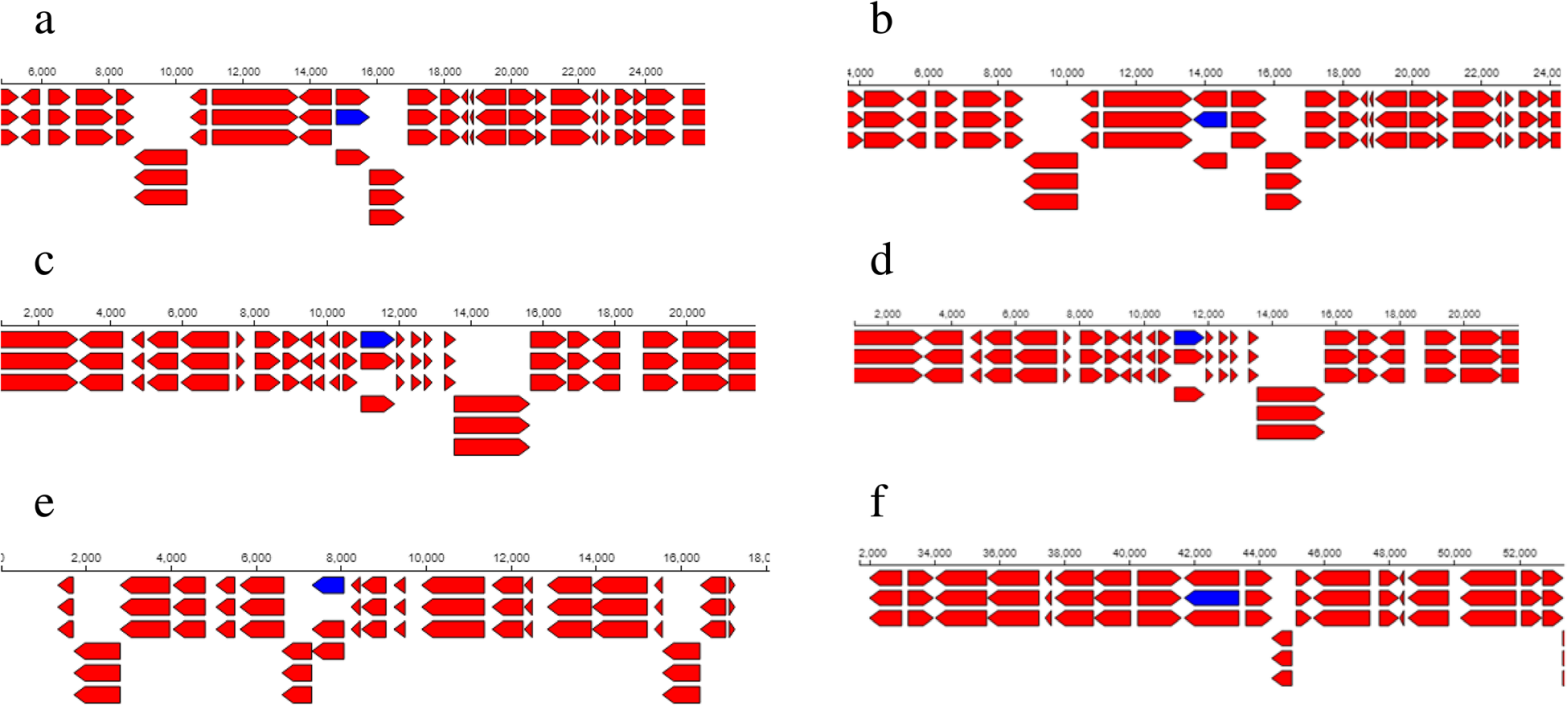
Feature context of **a** putative siderophore transport system permease protein YfiZ **b** putative siderophore-binding lipoprotein YfiY **c** Inner membrane protein YiaA **d** tRNA dimethylallyltransferase **e** Heptaprenyl diphosphate synthase component 1 **f** Septation ring formation regulator EzrA depicting gene names in the genome map of R3. The blue bars represent the gene locations

R1 and R3 strains were found to have 13 and 33 siderophore-producing genes respectively. To be specific, Catecholate siderophore receptor fiu and 2, 3-dihydro-2, 3-dihroxybenzoate dehydrogenase genes were noticed in R1 strain (Table 3) while putative siderophore transport system permease protein YfiZ and putative siderophore-binding lipoprotein YfiY genes were detected in R3 strain (Table 4). It is presumed that these siderophore protein-producing genes in R1 strain located between contigs 662–1,424 and 59–572 (Fig. 9a, b) and in R3 strain located between 14769 and 15,771 and 13,660–14,638 (Fig. 11a, b) could have contributed to the quantitative siderophore production (Fig. 2c) observed in this study, which can help these rhizobia strains to chelate Fe and make it available for plant use. Naturally, the role of siderophore producing-rhizobia under drought conditions is highly appreciated since it was reported by [65] that plants are highly prone to pathogenic attack under water stress conditions. Siderophore is obviously antimicrobial in nature [2] and thus application of siderophore-producing rhizobia will certainly help to improve plant health under drought stress and consequently increase agricultural productivity [66].

Furthermore, IAA genes involved in the production of Isoaspartyl peptidase protein was detected in R1 strain (Table 3). Similarly, 2 of the 6 IAA genes found in R3 strain are responsible for the production of inner membrane protein YiaA and tRNA dimethylallyltransferase (Table 4) and the locations of the respective genes were between 536 and 704 for the R1 strain (Fig. 9c, d) and between contigs 11,701–11,965 and 10,955–11,900 for the R3 strain (Fig. 11c, d).

Based on the results of the growth response to different environmental pH, R1 and R3 strains can be considered acid-tolerant strains since they were able to survive and grow at a low pH of 4. Again, upon application in the field, these strains may be confronted with acid stress in acidic soils and within the symbiosome. According to [1], the mechanisms involved in rhizobial survival and growth in acidic environments are yet to be understood. *EptA* genes coding for UDP-N-acetylmuramate-L-alanyl-gamma-D-glutamyl-meso-2,6-diaminoheptandioate ligase and Phosphoethanolamine transferase were found in R1 strains. [1] also found genes in *R. tropici* CIAT 899 and *Rhizobium* sp. PRF 81 coding for ‘putative lipid A Phosphoethanolamine transferase’ similar to that found in R1 strain in this study. In *Salmonella typhimurium* and *Escherichia coli*, *eptA* gene is activated under mildly acidic environments and it was reported that *eptA* imposed acid tolerance in *Shigella flexneri* 2a [67]. Also, *eptA* genes were reported in *R. rhizogenes* K84, sinorhizobia, agrobacteria, but not in other *Rhizobium* spp. [1].

## Conclusions

It was found that *Rhizobium* sp. strain R1, *Rhizobium tropici* strain R2, *Rhizobium cellulosilyticum* strain R3, *Rhizobium taibaishanense* strain R4 and *Ensifer meliloti* strain R5 isolated from Bambara groundnut rhizosphere possess the PGP traits considered in this study. In particular, these rhizobial strains produced EPS, ACC and in addition were able to survive and grow at a temperature of 45 °C and in an acidic condition with a pH of 4. Consequently, R1, R3 and R5 strains enhanced the germination of soybean seeds (PAN 1532 R) under drought condition imposed by 4% PEG; nevertheless, *Rhizobium* sp. strain R1 and *R. cellulosilyticum* strain R3 inoculations were able to improve seeds germination more than R5. Thus, genomic insights into *Rhizobium* sp. strain R1 and *R. cellulosilyticum* strain R3 revealed the presence of some genes with their respective proteins involved in symbiotic establishment, drought tolerance and plant growth promotion. In particular, *exoX*, *htrA*, *Nif*, *nodA*, *eptA*, *IAA* and siderophore-producing genes were found in the two rhizobial strains. Therefore, the possible PGP ability of these rhizobial strains can further be harnessed for biotechnological application in the field especially in semiarid and arid regions of the globe.

## Data Availability

The data for R1 strain are available in NCBI database under Bioproject number PRJNA496421, Biosample number SAMN10240937, SRA Accession number SRR8060784. Similarly data for R3 strain are available in NCBI database under Bioproject number PRJNA496421, Biosample number SAMN10245972, SRA Accession number SRR8061690.

## Abbreviations

ACC: 1-aminocyclopropane-1-carboxylate
ACS: Aminocyclopropane-1-carboxylate synthase
ANOVA: Analysis of Variance
BLAST: Basic Local Alignment Search Tool
CAS: Chrome azurol S
CFU: Coliform forming unit
DNA: Deoxyribonucleic acid
EPS: Exopolysaccharide
GC: Guanine cytosine
HSPs: Heat shock proteins
IAA: Indole-acetic-acid
KEGG: Kyoto encyclopedia of genes and genomes
LB: Luria Bertani
NCBI: National Centre for Biotechnology Information
O.D: Optical density
PCR: Polymerase chain reaction
PEG: Poly-ethylene glycol
PGP: Plant growth promoting
R1: *Rhizobium* sp. strain R1
R2: *Rhizobium tropici* strain R2
R3: *Rhizobium cellulosilyticum* strain R3
R4: *Rhizobium taibaishanense* strain R4
R5: *Ensifer meliloti* strain R5
RAST: rapid annotation using subsystem technology
rpm: Revolution per minute
SAS: Statistical Analysis System
SE: Standard error
